# A spatially explicit approach for identifying, prioritizing, and estimating costs associated with potential floodplain easements

**DOI:** 10.1002/jeq2.70157

**Published:** 2026-02-28

**Authors:** Kelsey D. Karnish, Emily K. Zimmerman, John C. Tyndall, William J. Beck, Sierra G. Geer

**Affiliations:** ^1^ Department of Natural Resource Ecology and Management Iowa State University Ames Iowa USA

## Abstract

Over the past 150 years, land‐use changes from native ecosystems to row crop corn and soybeans have negatively impacted a variety of ecosystem services across the US Corn Belt, including nutrient attenuation, water storage, and habitat. Restoring floodplains in agriculturally dominated landscapes through United States Department of Agriculture Natural Resource Conservation Service programs (e.g., Emergency Watershed Protection program) could offer disproportionate opportunities to positively enhance ecosystem service benefits, yet identifying and prioritizing opportunities for conservation practices, including floodplain easements, to maximize environmental outcomes and financial resources remain challenging. This research sought to identify, prioritize, and evaluate costs associated with potential floodplain easements across the state of Iowa. Leveraging key geospatial data and criteria, including proportion of field within the 2‐ and 5‐year flood return interval, field boundaries, and land use characteristics, we identified 141,314 floodplain easement opportunities of which 2707 were identified as high‐priority locations based on the inundation frequency of the fields, representing a total area of 56,300 ha. To estimate acquisition, direct, and total costs associated with potential easements, we used soil and crop productivity index information, land value data, historic vegetation information, and partial enterprise budgets. Estimated costs to restore only high‐priority easement opportunities totaled nearly 84.8 million USD annually. This approach aimed to develop an accessible approach to assist natural resource practitioners and conservation planners in identifying, prioritizing, and estimating costs for potential floodplain easement locations across the state of Iowa and in other regions where data exist.

## Core Ideas

- Suitable sites for floodplain easements were identified using geospatial technology.
- A prioritization score was assigned based on flooding frequency.
- Acquisition, direct, and total costs to restore suitable locations were estimated.
- The results aid in identifying high impact sites to effectively use limited funding.
- Restored floodplains provide habitat and attenuate nutrients and sediment.

## Plain Language Summary

Land‐use changes from native ecosystems to row crop corn and soybeans have negatively impacted downstream ecosystems across the United States (US) Corn Belt. Floodplain restoration could offer opportunities to positively enhance ecosystem service benefits. This research sought to identify, prioritize, and evaluate costs associated with potential floodplain easement locations in Iowa. Utilizing geospatial data, suitability criteria, and land use characteristics, we identified 2,707 high priority floodplain easement locations in Iowa. To estimate costs associated with potential easements, we used soil productivity information, land value data, and partial enterprise budgets. Estimated costs to restore high priority easement opportunities totaled nearly 84.8 million USD annually. This approach could assist conservation planners in identifying, prioritizing, and estimating costs of potential floodplain easement locations.

**Abbreviations**
ACPF: Agricultural Conservation Planning Framework
ACPF FiNRT: Agricultural Conservation Planning Framework Financial and Nutrient Reduction Tool
DEP: Daily Erosion Project
CSR2: Corn Suitability Rating (version 2)
EPA: Environmental Protection Agency
EWP: Emergency Watershed Protection
FEMA: Federal Emergency Management Agency
GARC: Geographic Area Rate Cap
GIS: Geospatial Information System
IDNR: Iowa Department of Natural Resources
MLRA: major land resource area
NRCS: Natural Resources Conservation Service
USDA: United States Department of Agriculture
WEPP: Water Erosion Prediction Project

## 1 INTRODUCTION

During the last 150 years, the US Corn Belt has transitioned from diverse, perennial prairie to hydraulically modified, monoculture row‐crop production (Brown & Schulte, 2011; Ramankutty & Foley, 1999). In the state of Iowa, for example, 99% of wetlands, marshes, and small streams were removed with subsurface tiles and ditch drainage (Schilling et al., 2012; Stone, 2000). This widespread adoption of subsurface tile drainage caused changes in watershed characteristics such as increased base flow in Iowa rivers which, coupled with anticipated increases in extreme precipitation events, increases flooding frequency and damaging and costly flood events across the US Corn Belt (Kunkel, 2003; Neri et al., 2019; Schilling & Helmers, 2008; Schilling & Libra, 2003; Shirzaei et al., 2021). Coupled with subsurface drainage and altered hydrology, the management of agricultural production in the US Corn Belt has evolved as a two‐crop system that is heavily reliant on synthetic fertilizers and whereby the vast majority of cropped soil is bare for half of the year—which has exacerbated nonpoint source pollution of nitrogen and phosphorus to surface waters (Baker et al., 2018; Jones et al., 2018; Porter et al., 2015; USEPA, 2007). Excess nitrate and phosphorus in surface waters has negative impacts on aquatic organisms and recreation (Brooks et al., 2015; D. Wolf et al., 2017), human health (e.g., Comly, 1945; Essien et al., 2022; Inoue‐Choi et al., 2014), and water quality more broadly (e.g., Jones et al., 2018; Robertson & Saad, 2013)—locally and regionally. To mitigate these issues, the US federal government has provided aid after natural disasters and conservation payments to landowners intended in‐part to facilitate conservation practice implementation to reduce nonpoint source pollution. Between 1995 and 2021, weather‐related disaster payments totaled $1.05 billion, and conservation payments to landowners from the United States Department of Agriculture (USDA) totaled $6 billion in Iowa alone (Environmental Working Group, 2021).

Restored floodplains offer multiple environmental benefits, especially in agricultural landscapes where challenges associated with flooding and nonpoint source nutrient pollution occur. Floodplain wetlands have been shown to reduce or delay downstream floods by increasing water storage, and during dry periods, floodplain wetlands preserve soil moisture by slowing water re‐entry into the channel (Bullock & Acreman, 2003). Restored and natural floodplains also reduce nonpoint source pollution by intercepting flood waters and reducing flood water velocity and allow the water to deposit sediment, carbon, and nutrients, namely nitrogen and phosphorus, to the floodplain (Gordon et al., 2020; Mitsch, 2005; Mitsch et al., 2001; Noe & Hupp, 2005; Silvan et al., 2004; Tschikof et al., 2022; Verhoeven et al., 2006; K. L. Wolf et al., 2013). Restored floodplains can be leveraged to achieve local nutrient reduction goals as they offer average nitrate reduction and total phosphorus reduction fractions at 64.2% and 26.5%, respectively (Gordon et al., 2020). Floodplain restoration can provide ecosystem services beyond nutrient and sediment reduction as they offer additional benefits to wildlife habitat and recreation (Grizzetti et al., 2019; Posthumus et al., 2010). Because of the promise of such outcomes, floodplain restoration has been a fundamental concept for water quality policy and restoration projects throughout Europe (Moss, 2007; Zerbe, 2023), Asia (Mei et al., 2023; J. Zhang et al., 2022), New Zealand (Abell et al., 2023), and the United States.

The USDA Natural Resources Conservation Service (NRCS) and other US federal and state agencies have aided agricultural landowners in implementing conservation practices to restore floodplains to address flooding, meet state and federal nutrient reduction goals, and deliver other ecosystem service benefits (Mississippi River/Gulf of Mexico Watershed Nutrient Task Force, 2008). The Emergency Watershed Protection (EWP) program is an easement program designed to protect and enhance floodplains and wetlands in the United States by purchasing the land and placing it in a perpetual floodplain easement held by the US through the Secretary of Agriculture, though limited property rights are maintained by the former landowner following restoration (USDA NRCS, 2022; USDA, 2018). The restoration process is often unique for each floodplain easement, but restoring native herbaceous and woody vegetation based on soils is a primary component of easement conservation plans with hydrologic alterations to reconnect the floodplain to the watershed in some cases (Adams, personal communication, 2022; Tenold‐Moretz, personal communication, 2022; Paulin, personal communication, 2022). While investment in the EWP program has varied over time, in late 2022, the USDA NRCS was awarded $918 million to subsidize watershed programs nationwide, including EWP, for the 2023 fiscal year, and the EWP was awarded $925 million from the Consolidated Appropriations Act to restore critical floodplains and wetlands throughout the United States (USDA, 2023, 2022b). Nevertheless, there has not historically been a systematic way to prioritize floodplain restoration opportunities or estimate general resource needs relative to available funding sources.

Identifying opportunities for agricultural floodplain restoration offers potentially high nutrient reduction, despite requiring restoration on only small percentage of land within the Mississippi River Basin (Mitsch et al., 2001), and has the potential to simultaneously offer other ecosystem service benefits (USDA, 2022a). Conservation planning in agricultural landscapes is increasingly making use of geospatial data and tools with the Geographic Information Systems (GIS) software to effectively identify and assess areas for resource concerns, conservation practice opportunities, and biophysical and financial outcomes across relevant spatial scales, from sub‐field to watersheds (e.g., Bravard et al., 2022; Tomer et al., 2013; Tomer, Porter, et al., 2015; Tomer, Boomer, et al., 2015). The Agricultural Conservation Planning Framework (ACPF), for example, is a decision support tool that utilizes geospatial datasets and tools to identify at watershed scales suites of field‐scale conservation practices that are biophysically suitable to address localized soil and water quality concerns (Porter et al., 2023; Tomer et al., 2013). The analytical results of the ACPF provide scenarios of opportunities to reference when natural resource practitioners and conservation specialists are consulting with landowners and farmers for practice implementation (Gesch et al., 2020; Lewandowski et al., 2020). To complement the outputs from the ACPF, the Financial and Nutrient Reduction Tool (FiNRT) was created to calculate associated short‐ and long‐term costs of conservation practices being considered as well as estimated nitrate reduction (Bravard et al., 2022). Presently, floodplain restoration practices, such as those offered via EWP, are not included in the ACPF or the ACPF FiNRT. Though there is software available to quantify resource concerns such as soil erosion (Daily Erosion Project [DEP], 2023; Flanagan & Nearing, 2000) and analyze watershed planning scenarios (e.g., the ACPF; Bravard et al., 2022; Porter et al., 2023; Tomer et al., 2013), a geospatial tool to site, prioritize, and evaluate costs of opportunities for floodplain restoration does not currently exist.

**Core Ideas**

- Suitable sites for floodplain easements were identified using geospatial technology.
- A prioritization score was assigned based on flooding frequency.
- Acquisition, direct, and total costs to restore suitable locations were estimated.
- The results aid in identifying high impact sites to effectively use limited funding.
- Restored floodplains provide habitat and attenuate nutrients and sediment.

This study built upon the ACPF and FiNRT frameworks to demonstrate the application of GIS‐based tool to identify, quantify, and prioritize suitable floodplain easement locations throughout the state of Iowa and to quantify the acquisition and direct costs associated with those floodplain restoration opportunities. Providing conservation agencies, allied nongovernmental organizations, and landowners with additional planning information, data, and certainty about conservation practices and their associated environmental and financial outcomes can positively influence adoption (Tyndall et al., 2013; Zimmerman et al., 2019). Moreover, given limited human and financial capital, identifying and prioritizing conservation opportunities that provide environmental benefits at relatively low costs are an increasingly important step in conservation planning (Zimmerman et al., 2019). The decision support tool and accompanying information presented in this study can help conservation planners and decision makers identify and prioritize sites for floodplain restoration to reduce nutrient and sediment load throughout the Mississippi River Basin and to the Gulf of Mexico and understand financial costs associated with potential floodplain easements in agricultural landscapes.

## 2 MATERIALS AND METHODS

### 2.1 Study location

For this study, a geospatial tool was created to identify suitable locations for floodplain easements based on NRCS criteria using flooding frequency, land use, and field boundary data, and suitable locations were assigned a prioritization score based on inundation frequency. Once suitable locations were identified, acquisition and direct costs associated with restoration were quantified for each identified floodplain easement opportunity. Costs were generated using soil productivity information, average land values, and specific direct cost associated with floodplain restoration actions. The US Corn Belt state of Iowa is dominated by row‐crop production; two‐thirds of Iowa is in corn and soybean production, producing an estimated $25 billion annually (United States Department of Agriculture National Agricultural Statistics Service, 2023, a map of the study location can be found in Figure S1). Iowa offers an opportune landscape to examine the potential for floodplain restoration via the EWP program because the land and hydrology have been heavily altered for row crop agriculture. Iowa leads the nation in Federal Emergency Management Agency (FEMA) disaster declarations for flooding (FEMA, 2024), and nonpoint source pollution from hydraulically altered agricultural landscapes in the state is impacting water quality, downstream ecosystems, and human health (e.g., Essien et al., 2022; Jones et al., 2018). The heavily altered agricultural landscape, multitude of resource concerns both locally and regionally, and large area make the state of Iowa a favorable study location to demonstrate a geospatial tool to site and prioritize floodplain easements and evaluate costs associated with floodplain easement implementation.

### 2.2 Identification and prioritization of potential floodplain easements

To identify suitable floodplain easements, we used the USDA NRCS criteria for EWP floodplain easements and applied the criteria at the field scale. According to USDA NRCS, fields that qualify for an EWP floodplain easement must be damaged by flooding at least two times within a 10‐year period, or once within the last 12 months, and the land must be privately owned (USDA, 2022a). Two primary use enrollment options are defined as (1) agricultural and open space or (2) residential. Fields with “residential” as the primary use must be part of a project to restore an entire floodplain reach with an appointed project sponsor (USDA, 2022a). For this research, which focused on floodplain easements as a tool to address environmental externalities associated with agricultural landscapes, we considered only the agricultural and open space use enrollment.

To identify suitable fields for potential EWP floodplain easement locations, we used geospatial data, including field boundaries, land use, and flood gradient data (Table 1). Statewide field boundary and land‐use data were taken from ACPF databases (Porter et al., 2023; Tomer et al., 2017). Land‐use data are derived from USDA CropScape Cropland Data Layer and summarized by field over a 6‐year period to identify a general land use (Tomer et al., 2017). To align with USDA NRCS qualifications, fields with row crop agriculture, pasture/grass/alfalfa, or forest were considered eligible for floodplain easements. The 2‐ and 5‐year flood gradient data layers were used to quantify the frequency of inundation for fields in Iowa (Iowa Department of Natural Resources [IDNR], 2021). The flood gradient data for Iowa have isolated gaps near two major rivers, the Mississippi and Missouri Rivers, which may have lowered the potential number of identifiable easements in our results (IDNR, 2021). Fields with eligible land use were evaluated based on inundation frequency utilizing the best available data, where suitable fields must intersect with the 5‐ or 2‐year flood frequency zone to meet the USDA NRCS inundation criteria.

**TABLE 1:** Data requirements for identifying and estimate costs for floodplain easements.

Data layer	Format	Spatial resolution (m)	Temporal resolution (year)	Description
Field boundaries	Polygon	0.0001	1 (2023)	Agricultural field boundaries manually updated from 2005/2008 USDA FSA data
Land use	Table	None	1 (average of 2018–2023)	Land use data derived from most recent 6 years of NASS Cropland Data Layers (CDL)
Flood gradient data	Polygon	0.0001	Single point in time	2‐ and 5‐year flood recurrence intervals produced by the Iowa Flood Center and Iowa DNR
Major land resource area boundaries	Polygon	2.22 × 10^−9^	Single point in time	Major land resource area boundaries for the United States, the Caribbean, and the Pacific Basin derived by the USDA NRCS
gSSURGO soil data	Raster	30	1 (2023)	Soil data for national, regional, and state use were created and maintained in USDA NRCS gridded soil survey geographic database
Iowa county boundaries	Polygon	0.0001	Single point in time	Statewide county boundary data created by and held in the Iowa Geospatial Data Clearinghouse
GLO historic vegetation data	Polygon	3.75 × 10^−9^	Single point in time	Digitized historic vegetation records from the GLO originally created between 1832 and 1859 and now held by the Iowa Geospatial Data Clearinghouse

*Note*: The data for field boundaries, land use, and grid scale soil information were taken from Agricultural Conservation Planning Framework (ACPF) database (Porter et al., 2023; Tomer et al., 2017).

Abbreviations: GLO, Government Land Office; NASS, National Agricultural Statistics Service; NASS, Natural Resources Conservation Service; USDA FSA, United States Department of Agriculture Farm Service Agency; gSSURGO, Gridded Soil Survey Geographic Database.

To prioritize floodplain easements, a weighted average scoring method was used to generate a suitability score based on inundation frequency. This scoring method is a multicriteria decision‐making method that produces a ranking and aggregated score, which can be used to compare sited opportunities (Jadhav & Sonar, 2009; Pérez & Rojas, 2000). First, the amount of the 2‐ and 5‐year flood frequency zone within each suitable field was calculated in area. To attribute a prioritization score to each field, more points were awarded to fields with larger proportions of high rates of inundation. An area‐weighted score was calculated based on the proportion of the field in the 2‐ and 5‐year flood frequency zone shown in Equation (1).

(1) $$\begin{array}{ccc} \mathrm{Score} & = & \left(\left(5\times 2\;\mathrm{yrArea}\right)+\left(2\times 5\;\mathrm{yrArea}\right)\right)\\ & & /\left(\mathrm{Total}\;\mathrm{Field}\;\mathrm{Area}\right). \end{array}$$

**FIGURE 1:**
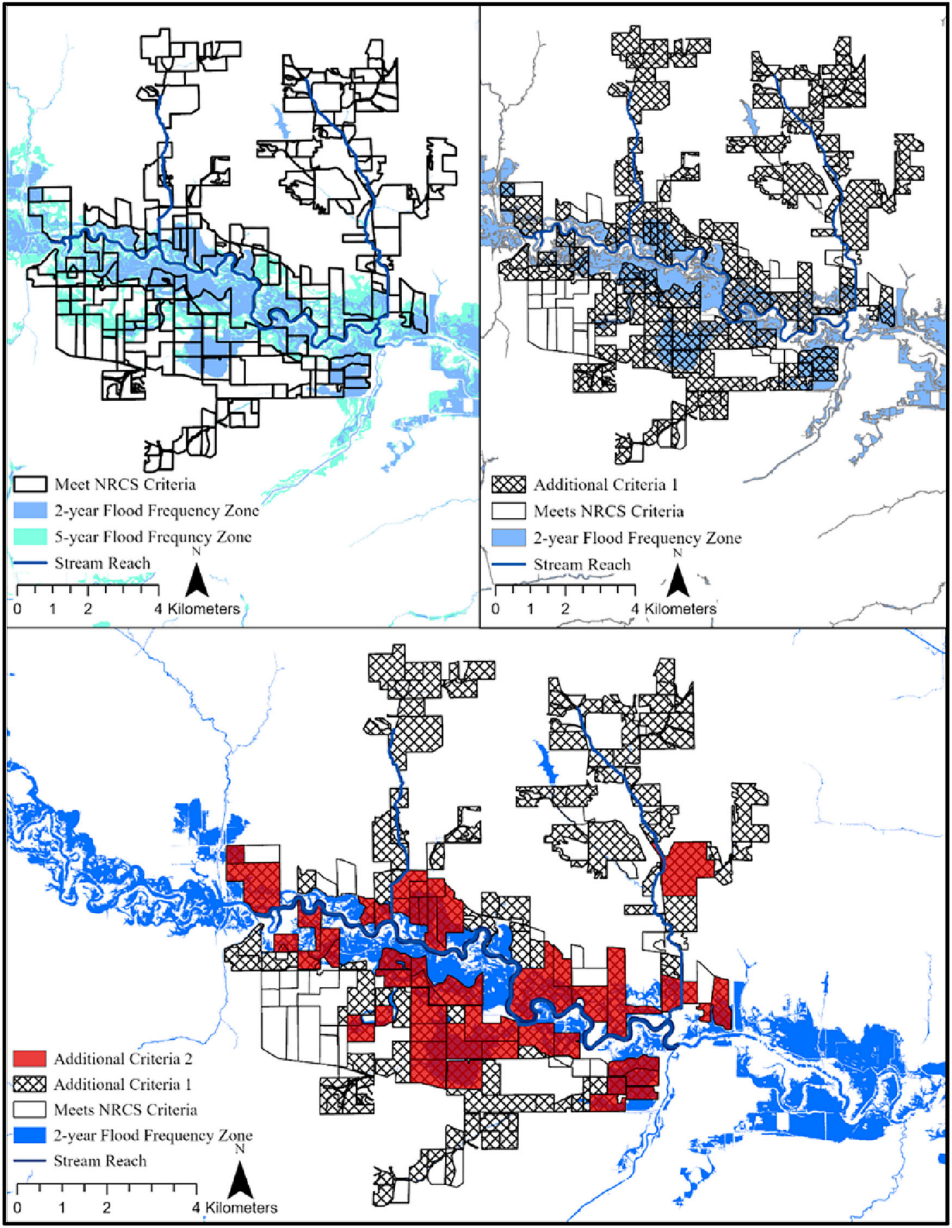
The tool created to identify and prioritize suitable fields for floodplain easements has options to add two additional criteria. Additional criteria are that (1) fields must intersect with the 2‐year flood frequency zone, and (2) the intersection with the 2‐year flood frequency zone must be greater than or equal to 2 ha.

An example of the score attribute calculation is presented in Figure S2. Scores range between 0 and 5 points, with 5 points indicating 100% of the field in the 2‐year flood frequency zone. The tool is compatible with the existing ACPF dataset and tool. An ArcGIS‐based toolbox and tool were created to automate this process. The tool includes an option to select two supplementary criteria that further limit results: (1) the site must intersect with the 2‐year flood frequency zone or (2) the site's intersection with the 2‐year flood frequency zone is greater than or equal to 2 ha. These options were added to restrict criteria further and offer increased practicality of the tool for conservation practitioners (Figure 1).

### 2.3 Acquisition and direct cost assessment of potential floodplain easements

Acquisition costs associated with removing agricultural land from production and placing it into a perpetual floodplain easement were calculated to satisfy USDA NRCS EWP program land acquisition guidelines. The USDA NRCS uses Geographic Area Rate Caps (GARCs) to appraise the value of the land dependent on the productivity of the land (USDA NRCS, 2023). The USDA NRCS, in consultation with the state technical committee, establishes regional GARCs using the best available information in the state, which may include data about soil types, types of crops capable of being grown, production history, location, real estate market values, and tax rates and assessments (Cornell Law School, n.d.). The GARC prices represent 85% of the fair market value (15% of the rights to the land are reserved for the landowner) and are generated and assigned as per acre values for five different regions in Iowa (USDA NRCS, 2023). Each region is assigned a price per acre for cropland and non‐crop land value, including forest, pasture, and grassland (USDA NRCS, 2023). Prices are assigned if area‐weighted Corn Suitability Ratings (version 2; CSR2) range between 55 and 83 (USDA NRCS, 2023). The CSR2 is a point‐based corn productivity index (1–100, where higher scores indicate higher productivity) unique to Iowa but broadly used throughout the state to classify and rank soil types. The CSR2 score is calculated using soil characteristics such as soil profile and slope (Miller & Burras, 2018). In Iowa, the GARCs range from $10,586 per hectare to $27,557 per hectare (USDA NRCS, 2023). If the area‐weighted CSR2 is outside of that range, an independent appraisal is required, and the GARC prices act as a “Not to Exceed” rate (USDA, 2023), meaning that the appraisal value is capped at the region‐specific GARC.

To determine if a potential floodplain easement would be eligible for the regional GARC or subject to an independent appraisal, we calculated area‐weighted CSR2 ratings and land values for each potential easement using the CSR2 and county‐level average land prices per acre. We adapted methods from Zimmerman et al. (2019) and Bravard et al. (2022) to correlate CSR2 values associated with soil map units in the gSSURGO soils database (Gridded Soil Survey Geographic Database) using custom extractions from the ACPF (Tomer et al., 2017; USDA, 2022c) and reported county‐level land value data (W. Zhang, 2022). County‐level data were aggregated at the major land resource area (MLRA) level. Iowa has 10 MLRAs that are areas with similar soils, climate, and vegetation or crop types and align with biophysical variability, as opposed to political county boundaries (USDA NRCS, 2022). The land value was determined by aggregating county average land value per hectare and CSR2 to create MLRA‐level average values. Then, for each eligible easement, for each soil unit present in the easement, we multiplied the CSR2 of each soil unit by the area occupied by that soil unit. We summed those values and divided the summation by the area to quantify the area‐weighted CSR2 for each easement. Following the method utilized by Bravard et al. (2022), we calculated land value per CSR2 point by dividing the average per hectare land value by the average CSR2 for the MLRA. The land value per CSR2 point was multiplied by the area‐weighted CSR2 for each easement to quantify by‐easement acquisition costs.

A final acquisition cost was assigned by comparing the value of each easement as quantified by the GARC versus independent appraisal and accounting for CSR2 and land use. If the field had an area‐weighted CSR2 between 55 and 83, the GARC‐determined value was assigned as the acquisition cost. If the CSR2 rating was greater than 83 or less than 55, the minimum price between the appraised and GARC cost was selected as the GARC price acts as a “Not to Exceed” price outside the range of the CSR2 range 55–83 (a demonstration can be found in Figure S3).

Direct costs for the different elements of floodplain easement restoration include site preparation; native seed, tree, and other vegetation stock; planting and establishment; and long‐term maintenance costs on a per hectare per year basis. To identify appropriate vegetation for various areas within each of the potential easements, each easement was delineated into various native land cover types (depressional wetlands, prairie, and forest) using information from the gSSURGO soils database and historical land cover information. Each soil map unit in the gSSURGO database is assigned a hydric rating. The hydric rating ranges from 0 to 100, where higher values indicate higher percentages of hydric soil conditions, and where, for example, species that tolerate wetter conditions may be more successful (Vasilas et al., 2010). To account for soil moisture and growing conditions, hydric categories were created mimicking a normal distribution and classified into four groups, each associated with different seed mixes and management for vegetation planting and wetland restoration: dry (hydric rating < 25), mesic (hydric rating < 25 and < 75), wet (hydric rating < 75 and < 100), and depression (hydric rating = 100). The area for each hydric rating category was then calculated within each potential easement. Dry, mesic, and wet categories indicate the type of native seed mix proposed to be planted in those areas, and the depression category indicates the area which should be restored as a depressional wetland. The area of historically present forest was also calculated at a per field basis to indicate the amount of forest present before European colonizers settled. Historically forested area was used to calculate the native riparian forest cover to restore within each easement opportunity at the field scale (Wilke‐Brown, 2017). Existing tree cover was not identified or considered when estimating costs for forest restoration.

To comprehensively account for the direct costs for restoring each hydric rating category and lands identified as historically forested, enterprise budgets were developed featuring a range of costs associated with site preparation, planting and establishment, and long‐term maintenance were determined using methods described in Tyndall and Roesch (2014). The budgets were used to calculate the present value costs of the different restoration options using standard discounted cash flow analysis over a 20‐year planning horizon using a 2% real discount rate in 2023$ (USD). A 2% real rate was utilized in accordance with similar water quality‐oriented assessments in Iowa as well as Environmental Protection Agency (EPA) guidelines (Bravard et al., 2022; USEPA, 2022; Hayes et al., 2016). Present value costs were then annualized using a capital recovery factor (Canada et al., 2003; a demonstration of cost calculations can be found in Figure S4; comprehensive budgets can be found in Tables S1–S4).

## 3 RESULTS

### 3.1 Identification and prioritization of potential floodplain easements

**FIGURE 2:**
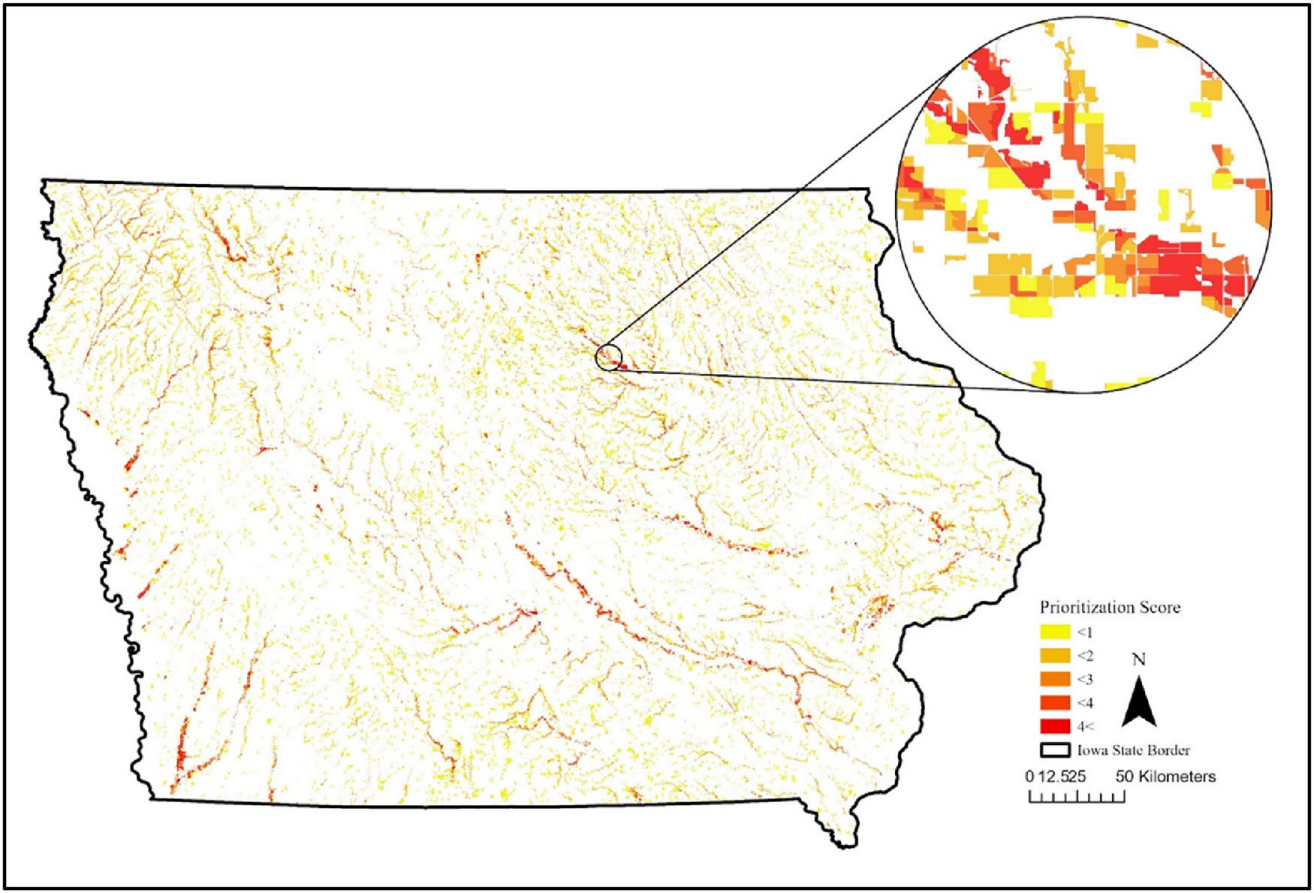
Suitable floodplain easement for Iowa classified by priority, where a higher score indicates higher priority.

Utilizing the floodplain easement identification and prioritization tool at the state level, 141,314 fields met the NRCS criteria and were identified as suitable for EWP floodplain easements. The suitable fields occupied 4,148,084 ha (29%) of the 14.6 million ha of Iowa. Eighty percent of these suitable fields (a total of 112,979 fields, covering 3.5 million ha), however, had a prioritization score less than 1, which indicated low priority for the floodplain easement opportunity. The average prioritization score from all identified sites was 0.64 out of a possible 5 points. With the additional criteria, which require the easement opportunity to intersect with the 2‐year flood frequency zone, 126,819 fields (3.8 million ha, 26% of state) were identified as suitable. Of the over 126,000 refined opportunities, ∼80% (101,211 fields) had a score less than 1, and the average score with this additional criterion was 0.65. Using both additional criteria, wherein the easement must overlap with at least 2 ha of the 2‐year floodplain, 36,066 fields (1,287,742 ha; 9% of the state) were identified as suitable for floodplain easements and had an average prioritization score of 1.64 (Figure 2). The application of increasingly restrictive criteria allowed for sites with greater areas of frequent inundation to be prioritized.

Of the 36,066 fields identified using both additional criteria, 21,742 fields were classified as agricultural land use, 5488 fields were in forest, and 8836 fields were classified as grass/pasture/alfalfa. Fields classified as agricultural occupied the greatest, 806,544 ha (Table 2). Prioritization scores given to eligible fields ranged from 0 to 5, where a score of 5 indicated the most frequent inundation. Relatively few fields (2707 fields, 4.4%) of those identified received prioritization scores greater than 4, indicating that a small number of fields were entirely within the 2‐year floodplain (Table S5). Fields with a prioritization score greater than 4 were categorized as high priority because of the high flooding frequency and assumed frequent interactions with flood waters. The tool not only demonstrates the efficacy of identifying suitable location for floodplain easements, but users can apply more restrictive criteria and assigned prioritization scores to further inform their site selection.

**TABLE 2:** Suitable floodplain easement numbers, areas, average prioritization score, and number and area of high‐priority easements by land use type for the state of Iowa, and annual total costs and per hectare average direct, acquisition, and total costs were calculated for all land use types.

Land use type	Number of suitable floodplain easements	Hectares of suitable floodplain easements (% total area), ha (%)	Average prioritization score	Number of suitable floodplain easements with score > 4	Hectares of suitable floodplain easements with score > 4 (% total area), ha (%)	Sum of total costs per year (2023 US$/year)	Mean direct costs per hectare per year (2023 US$/year)	Mean acquisition costs per hectare per year (2023 US$/year)	Mean total costs per hectare per year (2023 US$/year)
Forest	5488	213,332 (16.6)	1.41	138	1713 (0.13)	208,351,748	316	663	979
Agriculture	21,742	806,544 (62.6)	1.54	1632	36,492 (2.8)	1,386,762,537	274	1456	1730
Pasture	8836	267,866 (20.8)	2.05	937	18,123 (1.4)	219,770,760	310	560	870

*Note*: All cost information is presented in 2023 dollars.

### 3.2 Estimated acquisition, direct, and total costs of potential floodplain easements

Acquisition costs for restoring floodplain easement opportunities in Iowa which meet NRCS criteria and the intersection with the 2‐year flood frequency zone is at least 2 ha were estimated at over $1.5 billion per year. The mean annual acquisition costs per hectare was $1116, with the mean annual acquisition costs per suitable floodplain easement being $40,285 per year. Acquisition costs varied by land use, where the acquisition of agricultural land for floodplain easements (60% of the identified opportunities) was more than double the cost of non‐agricultural land at $1.2 billion per year ($1456 per hectare per year; Table 2). Acquisition costs for grass/pasture/alfalfa and forest, which represent the remaining 40% of the identified easement opportunities, were estimated to be over $281 million per year ($612 per hectare per year; Table 2). The sum of acquisition costs for high‐priority floodplain easement opportunities with prioritization scores greater than 4 was $60.2 million per year. The average acquisition cost per hectare per year was $1055, with the annual average acquisition cost per easement opportunity being $22,263.

Direct costs were estimated for restoration of dry, mesic, and wet prairie areas; depressional areas; and forest areas. Areas for potential floodplain restoration identified as dry, mesic, wet, depression, and forest were 824,058, 109,465, 204,276, 149,328, and 266,388 ha, respectively. Direct costs associated with restoration were assessed across a range of low, average, and high‐cost estimates to reflect likely variability in direct costs. Per hectare, annual average costs associated with restoration were the highest for depressional areas ($482; Table S6). Total average annual direct costs for all easement opportunities were just over $362 million. Direct costs for an individual easement opportunity ranged from $6527 to $13,905, with an average of $10,036 per year. The average annual direct cost per hectare throughout the state was $289 per hectare. In the aggregate, using average per hectare costs, fields with agricultural land use had the highest total annual direct costs at $214.7 million, which reflects that most easement opportunities (60%) intersect with agricultural land use. The average annual direct costs per hectare were the greatest on forested land at $316 (Table 2). Direct costs for restoring high‐priority floodplain easement opportunities totaled $24.6 million per year, with the average annual direct costs per hectare being $416, and average direct costs per easement being $9079 per year.

The total average costs (acquisition and direct costs) to restore all identified floodplain easements, which met additional criteria, totaled $1.8 billion per year. The average total cost for restoring a single floodplain easement opportunity in the state of Iowa was $50,321 per year, and the average per hectare cost was $1405 per year. The annual costs for restoring agricultural, grass/pasture/alfalfa, and forest totaling $1.4 billion, $220 million, and $208 million, respectively. The total costs per hectare were the highest when restoring agricultural land at $1730 per hectare per year (Table 2). For high‐priority floodplain easement opportunities, the total costs were $84.8 million per year, and the annual average total cost per easement and per hectare was $31341 and $1472, respectively. Acquisition, direct, and total costs for floodplain easement implementation in Iowa can be used to inform funding allocation for conservation practice implementation and adoption. When estimated costs are coupled with locations of high‐priority floodplain easement sites, conservation planners and allied watershed‐level organizations can better evaluate resource allocation needs and engage in a more comprehensive conversation with landowners.

## 4 DISCUSSION

This study demonstrated the application of geospatial data and the development of a GIS‐based tool to identify, quantify, and prioritize suitable floodplain easement locations throughout the state of Iowa and to quantify the annual acquisition and direct costs associated with those floodplain restoration opportunities. Using the most restrictive selection criteria, we identified 36,066 fields eligible for floodplain restoration in Iowa. Eligible fields occupied more than 1.2 million ha and had a mean prioritization score of 1.64; 2707 fields had a prioritization of greater than 4, indicating high‐priority locations for floodplain restoration. Total cost, meaning the sum of acquisition and direct costs, associated with restoring all eligible fields was estimated at $1.5 billion per year.

While more than 141,314 fields (4.1 million ha; 29% of Iowa) met the NRCS criteria and were identified as suitable for EWP floodplain easements, 80% of the identified fields had a prioritization score of less than 1, indicating low priority of floodplain easement restoration because of relatively small areas occupying the 2‐ and 5‐year flood gradient. This highlights the importance of utilizing the additional criteria of (1) ensuring the field intersects the 2‐year flood frequency zone and (2) the intersection with the 2‐year flood frequency zone is greater than or equal to 2 ha. The results from utilizing the most restrictive criteria are directly related to the 2‐year flood frequency zone, and in areas with a greater area of land in the 2‐year flood frequency zone, more high‐priority floodplain easement opportunities are anticipated. Because high‐priority fields, fields with a prioritization score greater than 4, largely reside within the 2‐year flood frequency zone, the count of opportunities with a score greater than 4 does not change drastically across criteria options. Adding these restrictive criteria increases the likelihood of more frequent flooding and thus more opportunities for the land, if restored, to carry out ecosystem services associated with restored wetland and forest floodplains (e.g., Mitsch et al., 2001; Noe & Hupp, 2005; Silvan et al., 2004; K. L. Wolf et al., 2013). Pragmatically, these restrictive criteria may also facilitate the directive of relatively limited conservation resources, including technical and financial assistance to priority regions and/or easements.

By intercepting flood waters, reducing flood water velocity, and attenuating sediment and nutrients to the floodplain, restored floodplains offer incredible opportunities in Iowa to achieve nutrient reduction goals provided by the Iowa Nutrient Reduction Strategy as floodplains offer average nitrate reduction and total phosphorus reduction fractions at 64.2% and 26.5%, respectively (Gordon et al., 2020; Mitsch, 2005; Mitsch et al., 2001; Noe & Hupp, 2005; Silvan et al., 2004; Tschikof et al., 2022; Verhoeven et al., 2006; K. L. Wolf et al., 2013). The data collected on floodplain easement efficacy within Iowa along the Iowa River suggest significant annual sediment (37,300 kg per hectare per year) and phosphorus (39,200 g per hectare per year) attenuation (Geer, 2024). Nitrate attenuation on existing floodplain easements in Iowa was found to be 200 g per hectare per day of a flooding event (Geer, 2024). If the total area of opportunities in Iowa that meet NRCS criteria and the intersection with the 2‐year flood frequency zone is at least 2 ha (1.29 million hectares) endured one flood event with a duration of 9.5 days (the average duration of floods), the floodplain opportunities would reduce 2,447 metric tons of nitrate from waterways throughout the state based on estimated nitrate attenuation data (Dartmouth Flood Observatory, 2004; Geer, 2024). If only the 2‐year flood frequency zone of the identified opportunities endured the same flooding conditions, the floodplain area would capture 487.6 metric tons of nitrate from flood waters throughout the state of Iowa in one flood event. Though these are preliminary findings, the nitrate, phosphorus, and sediment capture data from floodplains in Iowa demonstrate the opportunity potential floodplain easements offer for nutrient and sediment attenuation. The restoration of floodplain ecosystems offers additional opportunities for water storage, recreation, and increased habitat (Grizzetti et al., 2019; Jakubínský et al., 2021; Peh et al., 2014; Posthumus et al., 2010). The floodplain easement identification and prioritization tool and cost information in conjunction with nutrient attenuation and ecosystem service data can be leveraged to increase and ensure the cost‐effective implementation of floodplain easement opportunities.

Similar to other research about other best management practices (BMPs; e.g., Bravard et al., 2022), prioritizing floodplain easements for restoration based on flood frequency, and therefore likely nutrient reduction efficacy, would increase the cost‐effectiveness and potentially minimize the social and economic tradeoffs associated with restoration. For example, funding for floodplain easement restoration within the state of Iowa could be prioritized to regions, or even specific fields, with scores indicative of high frequencies of flood inundation to effectively reduce nitrate or deliver other ecosystem service benefits. This prioritized approach could minimize land conversion rates from row crop production to restored floodplain easements while maximizing water quality benefits derived from this practice. Moreover, concentrated conservation investments (e.g., awareness and education campaigns, technical assistance, financial resources) in priority areas have shown potential to accelerate adoption, particularly of edge of field and riparian practices (e.g., DeLong, 2023).

While this study highlights opportunities for identifying, prioritizing, and assessing costs associated with floodplain restoration in the US Corn Belt state of Iowa, this research has potential applications throughout the broader US Corn Belt. This research works to automate the process of identifying suitable locations for floodplain restoration, which provides a suite of benefits, namely the reduction in nutrients and sediment from waterways. Research worldwide has demonstrated the need to approach floodplain rehabilitation and management from a planning perspective, and as such the planning approach described here is relevant not only within North America (Llewellyn et al., 1996; Roley et al., 2012) but also internationally (e.g., Brunet & Austin, 2000; Garcia‐Linares et al., 2003; Halik et al., 2019; Li et al., 2009; Luke et al., 2017; Poesio et al., 2023). Across the United States, there are currently 1697 EWP program easements covering 76,201 ha, and federal funding initiatives aimed at conserving more critical wetlands and floodplains throughout the United States are expected to total over $1 billion (USDA, 2022b, 2023). The type of geospatial data required to run the GIS‐based tool developed in this study is generally available throughout the US Corn Belt and could be applied with adjustments. For example, while we used flood frequency data from the state of Iowa (IDNR, 2021) to identify and prioritize potential easements, the FEMA has existing nationwide flood hazard maps that could be evaluated for this application (FEMA, 2024), and several states have developed their own flooding inundation information. Similarly, land use data from the USDA CropScape Cropland Data Layer can be used to classify land use into agricultural, pasture, or forest. Specific data on cropping systems and land cover types would not be necessary, though current land management could impact desire for floodplain restoration projects in locations where floodplains and wetlands are under perennial cover already. The data required to assess acquisition and direct costs may be more challenging to identify and assemble. While the USDA National Commodity Crop Productivity Index is available nationally and provides a productivity index for various commodity crops, it is not intended to replace state‐specific productivity indices (e.g., CSR2) and may be more difficult to correlate to land market values (Albers et al., 2022).

This research relies on and is limited by the availability of high‐resolution geospatial data and could be enhanced with additional data. Prior research has demonstrated a strong correlation between nutrient attenuation and flooding inundation (Hefting et al., 2004). In this study, assumptions of flood frequency are based on general flood frequency gradients, not inundation period, and flood frequency gradients inform both suitability and prioritization of floodplain easement opportunities. The flood frequency data are not updated regularly to reflect areas that have flooded in the recent past, which could limit the accuracy of site selection. Integrating precise precipitation data could identify regions that are experiencing greater amounts of precipitation, and thus flooding. The Water Erosion Prediction Project (WEPP) Erosion Prediction Model integrates daily precipitation data to quantify daily erosion totals, and the DEP uses information both from WEPP and a Next‐Generation Weather Radar rainfall product to quantify soil erosion as well (DEP, 2023; Flanagan & Nearing, 2000). Though the output from these models yields soil loss data at the raster pixel scale and is very different from that of this research, utilizing precipitation data on shorter temporal scales would increase precision for identifying and prioritizing potential regions of the state for easements that offer nutrient attenuation, enhanced water storage, and decrease downstream flood intensity. Models of annual inundation extent could improve site selection and strengthen predictions of flooding occurrence by accurately identifying fields and proportions of fields that have flooded. Additionally, this tool identifies floodplain easement opportunities at the field scale, though the EWP program allows land to be enrolled at the subfield level. To narrow the selection of identified suitable floodplain easement locations, the flood frequency data could be leveraged to select only the land assumed to flood within the 2‐ and 5‐year flood frequency intervals to make the tool more precise in its selection of suitable land.

## 5 CONCLUSION

The principal objective of this research was to identify, prioritize, and estimate costs for suitable floodplain easement opportunities using geospatial data and tools to provide additional information for natural resource practitioners when communicating with landowners about prospective BMP implementation. The goals for this research were addressed by creating an automated suitability and ranking geospatial tool to identify and prioritize floodplain easement opportunities at the field and state scale and quantifying costs associated with floodplain easement land acquisition, restoration, and maintenance. Ultimately, this research provides natural resource practitioners and conservation specialists with an additional BMP identification tool and cost information to assist landowners and watershed stakeholders when considering BMP implementation, and the results can be leveraged to target cost‐effective high‐priority sites or regions for restoration.

## AUTHOR CONTRIBUTIONS

**Kelsey D. Karnish**: Data curation; formal analysis; methodology; validation; visualization; writing—original draft; writing—review and editing. **Emily K. Zimmerman**: Conceptualization; funding acquisition; methodology; supervision; writing—original draft; writing—review and editing. **John C. Tyndall**: Conceptualization; formal analysis; funding acquisition; writing—review and editing. **William J. Beck**: Data curation; methodology; writing—review and editing. **Sierra G. Geer**: Writing—review and editing.

## CONFLICT OF INTEREST STATEMENT

The authors declare no conflicts of interest.

## Supporting information



Supplementary Material
