# Supplementary material for: A spatially explicit approach for identifying, prioritizing, and estimating costs associated with potential floodplain easements
Source: J Environ Qual. 2026 Feb 28;55(2):e70157. doi: 10.1002/jeq2.70157 (PMC12949626; doi:10.1002/jeq2.70157)
Supplement: Supplementary file 1 — Supplementary Material [file JEQ2-55-0-s001.docx]

**Supplemental Materials:**


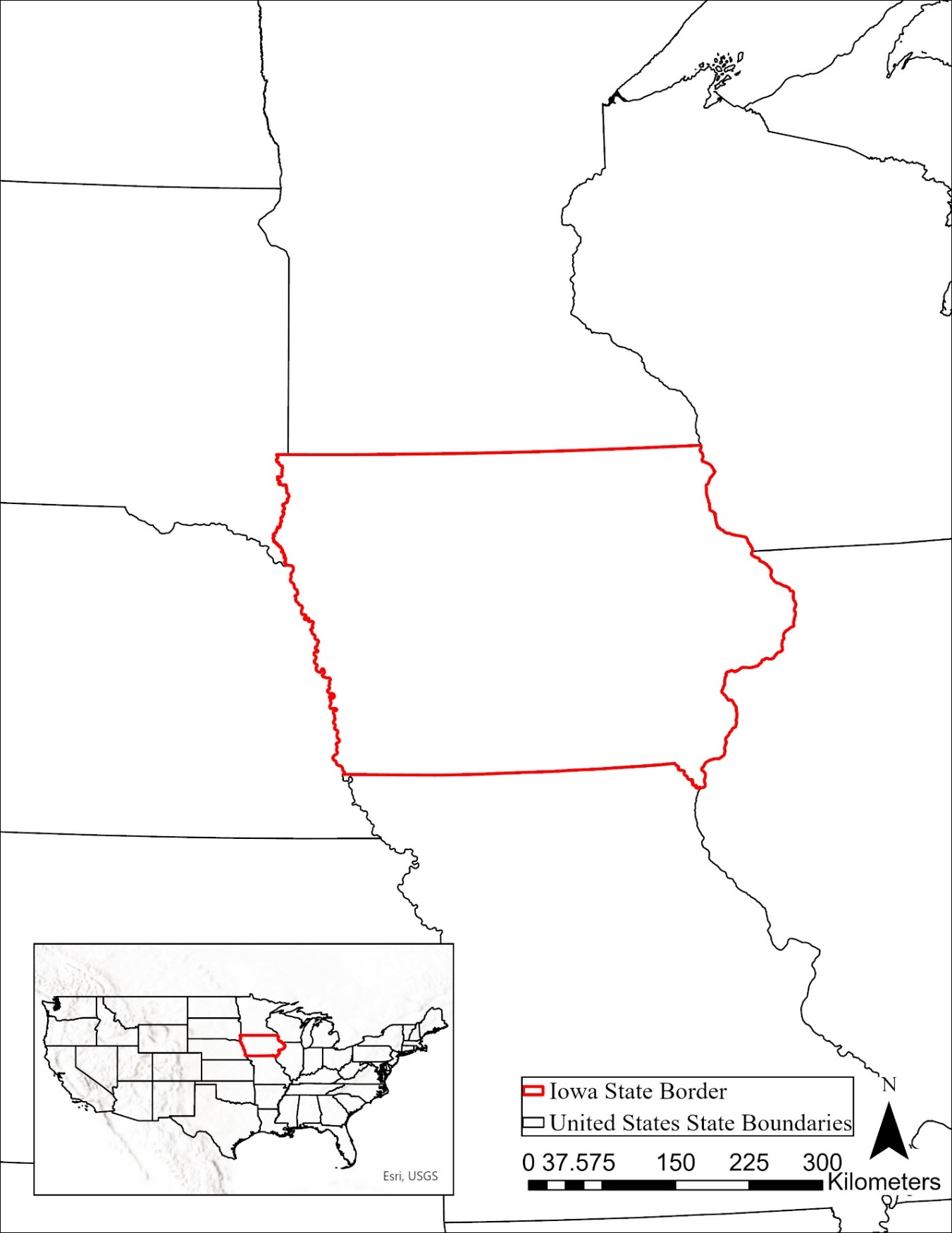


**Supplemental Figure S1.** Iowa is in the U.S. Corn Belt and is bordered by the states of Wisconsin, Illinois, Minnesota, Missouri, South Dakota, and Nebraska.


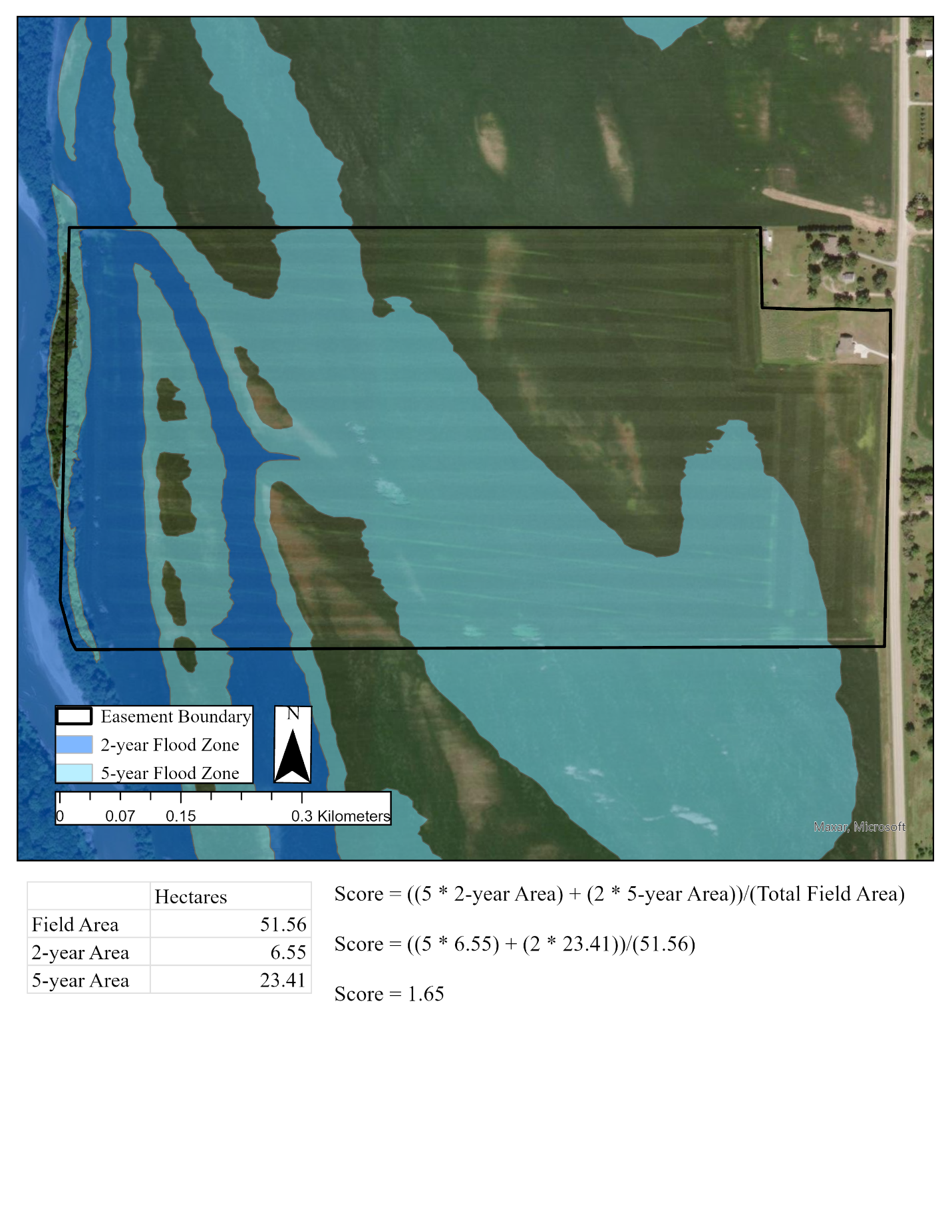


**Supplemental Figure S2.** An example of a prioritization score attribution calculation for a suitable field.


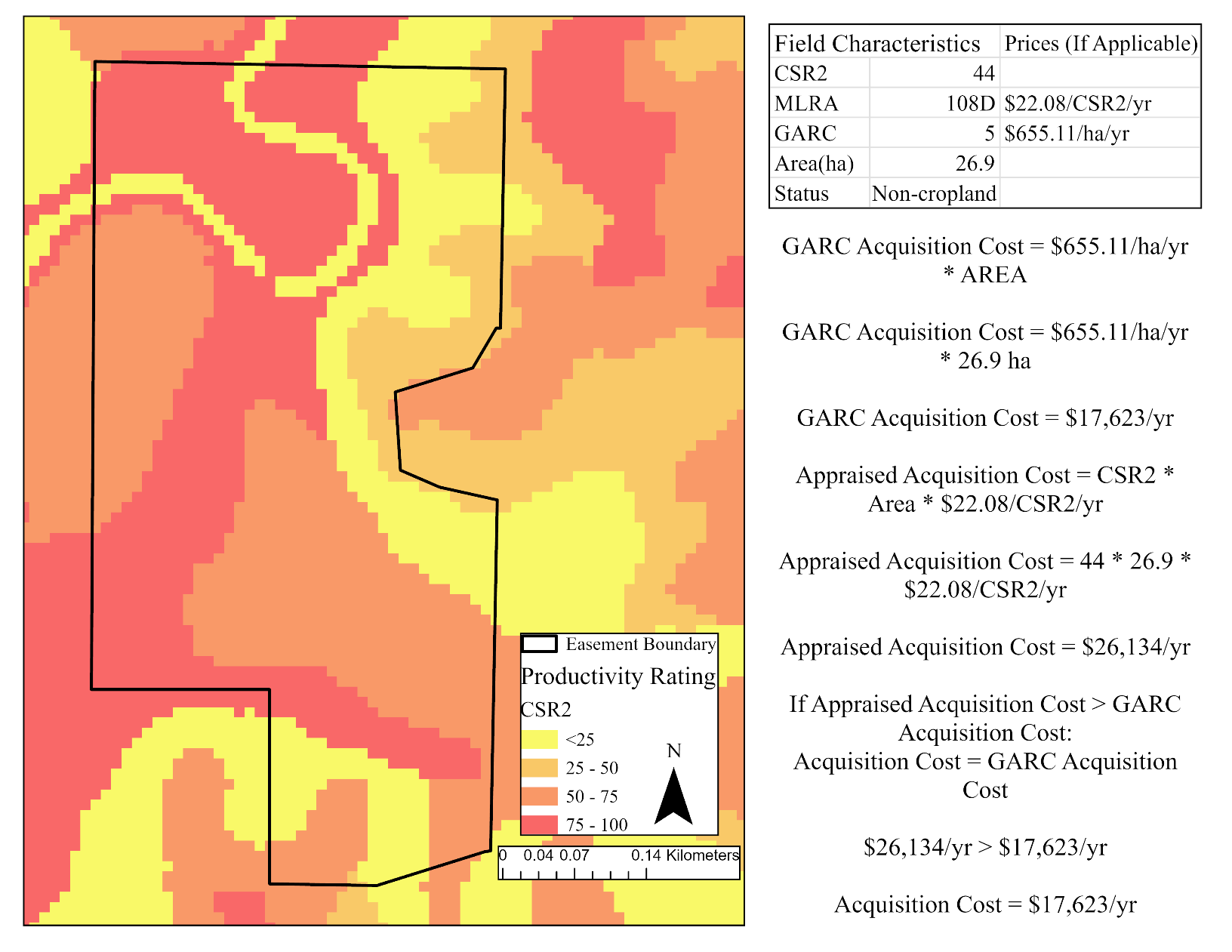


**Supplemental Figure S3.** The acquisition costs per year for each easement were calculated using soil productivity characteristics and per hectare costs associated with geographic location. For every potential easement location with an area weighted average CSR2 less than 55 or greater than 83, the GARC acquisition cost and appraisal cost was calculated, and the lower cost option was selected.


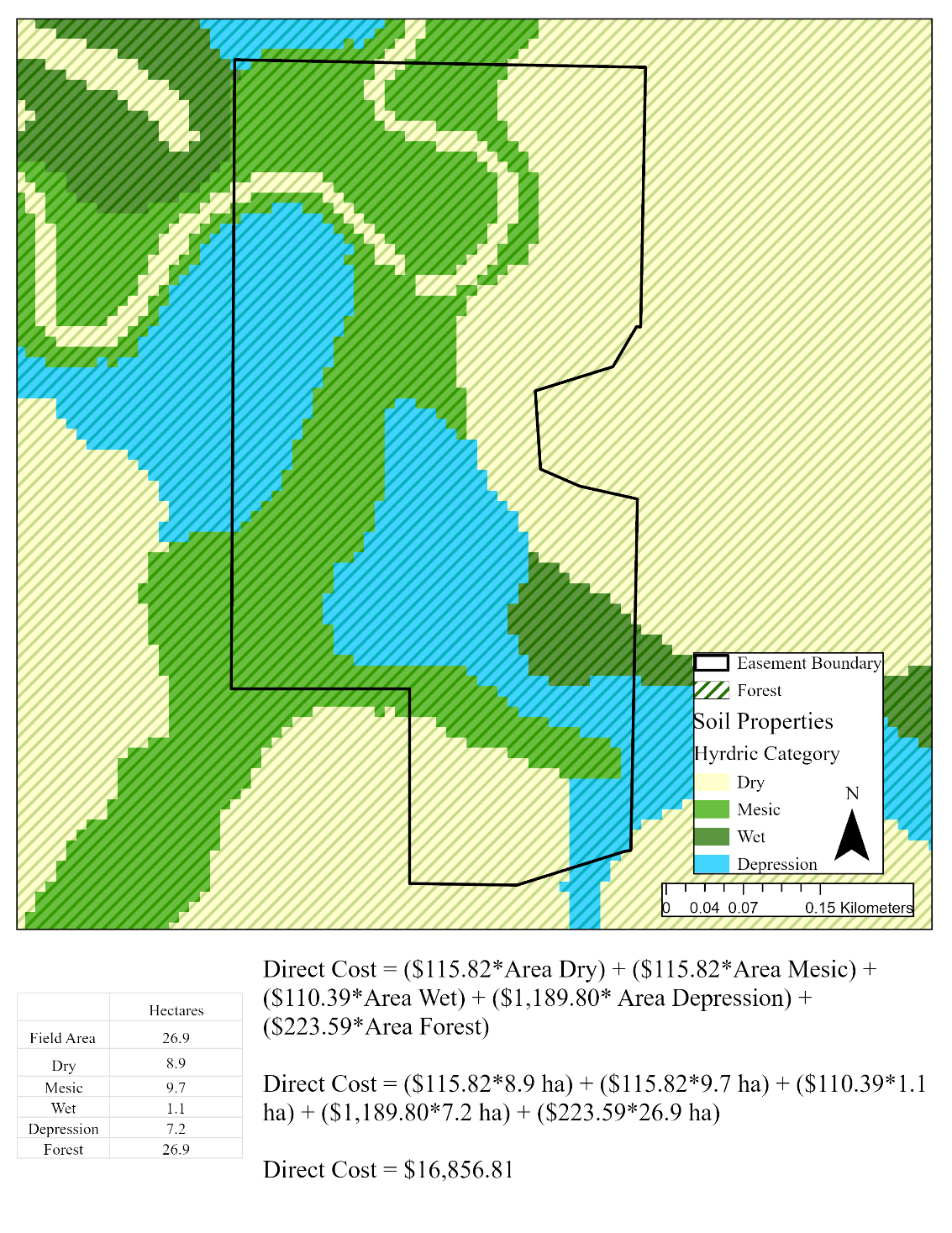


**Supplemental Figure S4.** The direct costs for each easement were calculated using spatially explicit soil characteristics about hydric properties, and seed mix costs were assigned based on hydric category. Dry was hydric < 25 , mesic was hydric > 25 and hydric < 75, wet was hydric < 75 and hydric < 100 , and wetland was hydric = 100. Soils data are from the gSSURGO data base (Soil Survey Staff, 2022), and were custom extracted for use with the field boundary data from the Agricultural Conservation Planning Framework (ACPF; Tomer et al. 2017).

**Supplemental Table S1.** Costs associated with planting seed mix in areas with dry hydric conditions. Costs presented in 2023 dollars.

| Establishment & Management | Cost Range LOW to HIGH $/acre | Cashflow LOW $/acre | Cashflow AVE $/acre | Cashflow HIGH $/acre | Year | Present Value LOW $/acre | Present value AVE $/acre | Present value HIGH $/acre | Annualized LOW $/acre | Annualized AVE $/acre | Annualized HIGH $/acre |
| --- | --- | --- | --- | --- | --- | --- | --- | --- | --- | --- | --- |
| Tillage | $11.00- $22.00 | $11.00 | $16.50 | $22.00 | 0 | $11.00 | $16.50 | $22.00 | $0.67 | $1.01 | $1.35 |
| Site prep for pasture | $10.00 to $17.00 x2 + $12.00 to $35.00 + $15.00 to $35.00 | $47.00 | $76.00 | $104.00 | 0 | $47.00 | $76.00 | $104.00 | $2.87 | $4.65 | $6.36 |
| Herbicide & application | $10.00 - $17.00 | $10.00 | $13.50 | $17.00 | 0 | $10.00 | $13.50 | $17.00 | $0.61 | $0.83 | $1.04 |
| Seed Drilling | $10.00 - $29.00 | $10.00 | $19.50 | $29.00 | 0 | $10.00 | $19.50 | $29.00 | $0.61 | $1.19 | $1.77 |
| Seed (variable; depends on site conditions) ^1^ | $156.00 | $156.00 | $156.00 | $156.00 | 0 | $156.00 | $156.00 | $156.00 | $9.54 | $9.54 | $9.54 |
| Cultipacking | $6.00 - $34.00 | $6.00 | $20.00 | $34.00 | 0 | $6.00 | $20.00 | $34.00 | $0.37 | $1.22 | $2.08 |
| Mowing | $10.00 - $50.00 | $30.00 | $90.00 | $150.00 | 1 | $29.41 | $88.24 | $147.06 | $1.80 | $5.40 | $8.99 |
| Raking | $5.00 - $15.00 | $15.00 | $30.00 | $45.00 | 1 | $14.71 | $29.41 | $44.12 | $0.90 | $1.80 | $2.70 |
| Mowing | $10.00 - $50.00 | $20.00 | $60.00 | $100.00 | 2 | $19.22 | $57.67 | $96.12 | $1.18 | $3.53 | $5.88 |
| Raking | $5.00 - $15.00 | $10.00 | $20.00 | $30.00 | 2 | $9.61 | $19.22 | $28.84 | $0.59 | $1.18 | $1.76 |
| Bump seeding | $85.00 | $85.00 | $85.00 | $85.00 | 3 | $80.10 | $80.10 | $80.10 | $4.90 | $4.90 | $4.90 |
| Management - Mow | $0.00 | $0.00 | $0.00 | $0.00 | every 5 |  |  |  | $0.00 | $0.00 | $0.00 |
| Management Burn | $13.00 - $60.00 | $52.00 | $148.00 | $240.00 | every 5 | $40.85 | $116.26 | $188.52 | $2.50 | $7.11 | $11.53 |
| Annual costs |  |  |  |  |  |  |  |  | $0.00 | $0.00 | $0.00 |
| Overhead | $2.16 - $8.91 | $2.16 | $5.54 | $8.91 | Annual | $35.32 | $90.51 | $145.69 | $2.16 | $5.54 | $8.91 |
|  |  |  |  |  |  |  |  | Cropped ground | $25.82 | $43.23 | $60.45 |
|  |  |  |  |  |  |  |  | Pasture ground | $28.02 | $46.87 | $65.46 |
| 1. Seed costs derived from the 2023 Iowa Pheasants Forever Native Seed Program: https://www.pfhabitatstore.com/store/items/IA/ | | | | | | | | |  |  |  |

**Supplemental Table S2.** Costs associated with planting seed mix in areas with mesic hydric conditions. Costs presented in 2023 dollars.

| Establishment & Managment | Cost Range LOW to HIGH $/acre | Cashflow LOW $/acre | Cashflow AVE $/acre | Cashflow HIGH $/acre | Year | Present Value LOW $/acre | Present value AVE $/acre | Present value HIGH $/acre | Annualized LOW $/acre | Annualized AVE $/acre | Annualized HIGH $/acre |
| --- | --- | --- | --- | --- | --- | --- | --- | --- | --- | --- | --- |
| Tillage | $11.00- $22.00 | $11.00 | $16.50 | $22.00 | 0 | $11.00 | $16.50 | $22.00 | $0.67 | $1.01 | $1.35 |
| Site prep for pasture | $10.00 to $17.00 x2 + $12.00 to $35.00 + $15.00 to $35.00 | $47.00 | $76.00 | $104.00 | 0 | $47.00 | $76.00 | $104.00 | $2.87 | $4.65 | $6.36 |
| Herbicide & application | $10.00 - $17.00 | $10.00 | $13.50 | $17.00 | 0 | $10.00 | $13.50 | $17.00 | $0.61 | $0.83 | $1.04 |
| Seed Drilling | $10.00 - $29.00 | $10.00 | $19.50 | $29.00 | 0 | $10.00 | $19.50 | $29.00 | $0.61 | $1.19 | $1.77 |
| Seed (variable; depends on site conditions) **^1^** | $120.00 - $192.00 | $120.00 | $156.00 | $192.00 | 0 | $120.00 | $156.00 | $192.00 | $7.34 | $9.54 | $11.74 |
| Cultipacking | $6.00 - $34.00 | $6.00 | $20.00 | $34.00 | 0 | $6.00 | $20.00 | $34.00 | $0.37 | $1.22 | $2.08 |
| Mowing | $10.00 - $50.00 | $30.00 | $90.00 | $150.00 | 1 | $29.41 | $88.24 | $147.06 | $1.80 | $5.40 | $8.99 |
| Raking | $5.00 - $15.00 | $15.00 | $30.00 | $45.00 | 1 | $14.71 | $29.41 | $44.12 | $0.90 | $1.80 | $2.70 |
| Mowing | $10.00 - $50.00 | $20.00 | $60.00 | $100.00 | 2 | $19.22 | $57.67 | $96.12 | $1.18 | $3.53 | $5.88 |
| Raking | $5.00 - $15.00 | $10.00 | $20.00 | $30.00 | 2 | $9.61 | $19.22 | $28.84 | $0.59 | $1.18 | $1.76 |
| Bump seeding | $85.00 | $85.00 | $85.00 | $85.00 | 3 | $80.10 | $80.10 | $80.10 | $4.90 | $4.90 | $4.90 |
| Management - Mow |  |  |  |  |  |  |  |  | $0.00 | $0.00 | $0.00 |
| Management Burn | $13.00 - $60.00 | $52.00 | $148.00 | $240.00 | every 5 | $40.85 | $116.26 | $188.52 | $2.50 | $7.11 | $11.53 |
| Annual costs |  |  |  |  |  |  |  |  | $0.00 | $0.00 | $0.00 |
| Overhead | $2.16 - $8.91 | $2.16 | $5.54 | $8.91 | Annual | $35.32 | $90.51 | $145.69 | $2.16 | $5.54 | $8.91 |
|  |  |  |  |  |  |  |  | Cropped ground | $23.62 | $43.23 | $62.65 |
|  |  |  |  |  |  |  |  | Pasture ground | $25.82 | $46.87 | $67.67 |
| ^1.^ Seed costs derived from the 2023 Iowa Pheasants Forever Native Seed Program: https://www.pfhabitatstore.com/store/items/IA/ | | | | | | | | |  |  |  |

**Supplemental Table S3.** Costs associated with planting seed mix in areas with wet hydric conditions. Costs presented in 2023 dollars.

| Establishment & Management | Cost Range LOW to HIGH $/acre | Cashflow LOW $/acre | Cashflow AVE $/acre | Cashflow HIGH $/acre | Year | Present Value LOW $/acre | Present value AVE $/acre | Present value HIGH $/acre | Annualized LOW $/acre | | Annualized AVE $/acre | | Annualized HIGH $/acre | |  |
| --- | --- | --- | --- | --- | --- | --- | --- | --- | --- | --- | --- | --- | --- | --- | --- |
| Tilling | $11.00- $22.00 | $11.00 | $16.50 | $22.00 | 0 | $11.00 | $16.50 | $22.00 | $0.67 | | $1.01 | | $1.35 | |  |
| Site prep for pasture | $10.00 to $17.00 x2 + $12.00 to $35.00 + $15.00 to $35.00 | $47.00 | $76.00 | $104.00 | 0 | $47.00 | $76.00 | $104.00 | $2.87 | | $4.65 | | $6.36 | |  |
| Herbicide & application | $10.00 - $17.00 | $10.00 | $13.50 | $17.00 | 0 | $10.00 | $13.50 | $17.00 | $0.61 | | $0.83 | | $1.04 | |  |
| Seed Drilling | $10.00 - $29.00 | $10.00 | $19.50 | $29.00 | 0 | $10.00 | $19.50 | $29.00 | $0.61 | | $1.19 | | $1.77 | |  |
| Seed (variable; depends on site conditions) **^1.^** | $138.00 | $138.00 | $138.00 | $138.00 | 0 | $138.00 | $138.00 | $138.00 | $8.44 | | $8.44 | | $8.44 | |  |
| Cultipacking | $6.00 - $34.00 | $6.00 | $20.00 | $34.00 | 0 | $6.00 | $20.00 | $34.00 | $0.37 | | $1.22 | | $2.08 | |  |
| Mowing | $10.00 - $50.00 | $30.00 | $90.00 | $150.00 | 1 | $29.41 | $88.24 | $147.06 | $1.80 | | $5.40 | | $8.99 | |  |
| Raking | $5.00 - $15.00 | $15.00 | $30.00 | $45.00 | 1 | $14.71 | $29.41 | $44.12 | $0.90 | | $1.80 | | $2.70 | |  |
| Mowing | $10.00 - $50.00 | $20.00 | $60.00 | $100.00 | 2 | $19.22 | $57.67 | $96.12 | $1.18 | | $3.53 | | $5.88 | |  |
| Raking | $5.00 - $15.00 | $10.00 | $20.00 | $30.00 | 2 | $9.61 | $19.22 | $28.84 | $0.59 | | $1.18 | | $1.76 | |  |
| Bump seeding | $66.00 | $66.00 | $66.00 | $66.00 | 3 | $62.19 | $62.19 | $62.19 | $3.80 | | $3.80 | | $3.80 | |  |
| Management - Mow | $0.00 | $0.00 | $0.00 | $0.00 | every 5 | $0.00 | $0.00 | $0.00 | $0.00 | | $0.00 | | $0.00 | |  |
| Management Burn | $13.00 - $60.00 | $52.00 | $148.00 | $240.00 | every 5 | $40.85 | $116.26 | $188.52 | $2.50 | | $7.11 | | $11.53 | |  |
| Annual costs |  |  |  |  |  |  |  |  | $0.00 | | $0.00 | | $0.00 | |  |
| Overhead | $2.16 - $8.91 | $2.16 | $5.54 | $8.91 | Annual | $35.32 | $90.51 | $145.69 | $2.16 | | $5.54 | | $8.91 | |  |
|  |  |  |  |  |  |  |  | Cropped ground | $23.63 | | $41.04 | | $58.25 | |  |
|  |  |  |  |  |  |  |  | Pasture ground | $25.83 | | $44.67 | | $63.27 | |  |
| **^1.^** Seed costs derived from the 2023 Iowa Pheasants Forever Native Seed Program: https://www.pfhabitatstore.com/store/items/IA/ | | | | | | | | | |  | |  | |  | |

**Supplemental Table S4.** Costs associated with establishing forests in areas identified as historically forested. Costs presented in 2023 dollars.

| **Cost Activities** | **Year cost incurred** | **Range of costs/acre** | **Low price** | **Mean price** | **High price** | **Present Value LOW $/acre** | **Present value AVE $/acre** | **Present value HIGH $/acre** | **Annualized LOW $/acre** | **Annualized AVE $/acre** | **Annualized HIGH $/acre** |
| --- | --- | --- | --- | --- | --- | --- | --- | --- | --- | --- | --- |
| Easement Site design | 0 | $0 to $500/ design | $- | $- | $500.00 | $- | $- | $500.00 | $0.00 | $0.00 | $30.58 |
| Site prep for pasture conditions | 0 | $10.00 to $17.00 x2 + $12.00 to $35.00 + $15.00 to $35.00/ acre | $107.00 | $136.00 | $164.00 | $107.00 | $136.00 | $164.00 | $6.54 | $8.32 | $10.03 |
| Site prep for cropland conditions | 0 | $140.00 to 380.00/acre | $140.00 | $227.00 | $380.00 | $140.00 | $227.00 | $380.00 | $8.56 | $13.88 | $23.24 |
| Tree stock (bare root seedlings) | 0 | $1.00/seedling @ 1200 to 1600/acre | $1,400.00 | $1,400.00 | $1,400.00 | $1,400.00 | $1,400.00 | $1,400.00 | $85.62 | $85.62 | $85.62 |
| Tree stock shipping | 0 | $50 per 500 seedlings | $140.00 | $140.00 | $140.00 | $140.00 | $140.00 | $140.00 | $8.56 | $8.56 | $8.56 |
| Tree planting cost | 0 | $100 to $427 | $100.00 | $264.00 | $427.00 | $100.00 | $264.00 | $427.00 | $6.12 | $16.15 | $26.11 |
| Pre-emergent Herbicide | 1 | $40 to $50/gal | $40.00 | $45.00 | $50.00 | $39.22 | $44.12 | $49.02 | $2.40 | $2.70 | $3.00 |
| Mortality replacement | 3 | $1.00 per seedling; 140 seedlings per acre | $140.00 | $140.00 | $140.00 | $131.93 | $131.93 | $131.93 | $8.07 | $8.07 | $8.07 |
| Planting replacement trees | 3 | $2.00 per tree | $280.00 | $280.00 | $280.00 | $263.85 | $263.85 | $263.85 | $16.14 | $16.14 | $16.14 |
| Herbicide application | 1,2,3,4,5 | $5.00 to $8.50/ acre | $5.00 | $7.40 | $9.00 | $23.57 | $34.88 | $42.42 | $1.44 | $2.13 | $2.59 |
| Herbicide | 2,3,4 | $15.00/ acre | $10.00 | $15.00 | $20.00 | $47.13 | $45.00 | $94.27 | $2.88 | $2.75 | $5.77 |
| Mowing cost | 1,2,3,4,5 | $20 to $60 / acre | $20.00 | $40.00 | $60.00 | $94.27 | $200.00 | $282.81 | $5.77 | $12.23 | $17.30 |
| **Annual costs** |  |  |  |  |  |  |  |  |  |  |  |
| Overhead | Annual |  | $53.40 | $60.93 | $85.41 | $873.17 | $996.29 | $1,396.58 | $53.40 | $60.93 | $85.41 |
|  |  |  | Total Present Value/ acre | $3,360.13 | $3,883.07 | $5,271.87 | $205 | $237 | $322 |  |  |
|  |  |  |  |  |  |  |  |  | **Annualized LOW $/acre** | **Annualized AVE $/acre** | **Annualized HIGH $/acre** |
|  |  |  |  |  |  |  |  | Cropped Ground | $198.95 | $229.16 | $312.38 |
|  |  |  |  |  |  | Pasture Ground | $196.93 | $223.59 | $299.17 |  |  |
|  |  |  |  | | |  |  |  |  |  |  |

| **Land Use Type** | **Average Prioritization Score** | **Number of Suitable Floodplain Easement Opportunities** | **Hectares of suitable floodplain easements** | **Score <1 Count** | **Score <1 ha** | **Score <2 count** | **Score <2 ha** | **Score <3 count** | **Score<3 ha** | **Score <4 count** | **Score<4 ha** | **Score 4=< count** | **Score 4=< ha** |
| --- | --- | --- | --- | --- | --- | --- | --- | --- | --- | --- | --- | --- | --- |
| Forest | 1.41 | 5,488 | 213,331.54 | 2,429.00 | 148,568.32 | 1,673.00 | 40,464.76 | 898.00 | 17,017.28 | 350.00 | 5,568.13 | 138.00 | 1,713.05 |
| Agriculture | 1.54 | 21,742 | 806,543.49 | 10,245.00 | 506,108.89 | 5,500.00 | 160,212.67 | 2,740.00 | 68,295.87 | 1,625.00 | 35,433.34 | 1,632.00 | 36,492.72 |
| Pasture | 2.05 | 8,836 | 267,865.43 | 2,367.00 | 131,904.76 | 2,411.00 | 59,073.63 | 1,866.00 | 34,816.48 | 1,255.00 | 23,947.28 | 937.00 | 18,123.29 |
| Total |  | 36,066.00 | 1,287,740.47 | 15,041 | 786,581.96 | 9,584 | 259,751.06 | 5,504 | 120,129.63 | 3,230 | 64,948.75 | 2,707 | 56,329.06 |

**Supplemental Table S5.** Suitable floodplain easement numbers, areas, average prioritization score, and number and area by prioritization score by land use type for the state of Iowa.

| **Land Condition** | **Annual Low Restoration Cost per Hectare** | **Annual Average Restoration Cost per Hectare** | **Annual High Restoration Cost per Hectare** |
| --- | --- | --- | --- |
| Dry Prairie | $64/ha | $107/ha | $149/ha |
| Mesic Prairie | $58/ha | $107/ha | $155/ha |
| Wet Prairie | $58/ha | $101/ha | $144/ha |
| Depressional | $296/ha | $482/ha | $667/ha |
| Forested | $199/ha | $229/ha | $312/ha |

**Supplemental Table S6.** The per hectare annual direct costs for restoration of dry, mesic, wet, depression, and forested land conditions for the state of Iowa. All cost information is presented in 2023 dollars.
